# Evaluation of articles in metabolism research on the basis of their citations

**DOI:** 10.11613/BM.2021.010201

**Published:** 2020-12-15

**Authors:** Yi Xiang Zhan, D’arcy Turner, Daniela Tritz, Kelly Natarajan, Mo Som, Matt Vassar

**Affiliations:** 1 Department of Research, Kansas City University of Medicine and Biosciences, Kansas City, USA; 2 Department of Psychiatry and Behavioral Sciences, Oklahoma State University, Tulsa, USA

**Keywords:** metabolism, research waste, Scopus, citation, bibliometrics

## Abstract

**Introduction:**

The number of research papers and journals each year is increasing and millions of dollars are spent. Despite this there is evidence to suggest that many publications do not impact clinical practice. We used citation analysis to measure the influence of metabolism publications from 2003-2013. Those papers with lower citation rates are likely to be of the least value and high rates of such publications may be a marker of research waste.

**Materials and methods:**

We analysed 67 journals with 81,954 articles related to metabolism indexed on the Scopus station database from 2003-2013. We identified those articles with less than 5 citations within 5 years from publication date as poorly cited. Journals were ranked by the percentage of articles that were poorly cited or uncited.

**Results:**

Over the 10-year period, the number of total articles increased by 127%. We found that 24% of articles were poorly cited within 5 years of publication. Journals in the bottom 25% and top 25% of rankings by citation rates accounted for a similar proportion of poorly cited articles. Most of the open access journals were ranked in the top 25% for citation rates.

**Conclusions:**

Our analysis contradicts concerns over increasing amounts of publications with little impact. The proportion of poorly cited articles are low, with little change in the trend over 10 years. The top and bottom ranked journals produced similar proportions of poorly cited articles. These findings suggest the necessity of pursuing further research to study waste in metabolism research.

## Introduction

The metabolism literature continues to grow every year. Concurrently, the greater research community has begun to describe a large body of research that may be “wasteful”. It is estimated that approximately 85% of health research is wasted, which would suggest that $170 of the $200 billion spent annually is on wasteful research (*1*). Research waste results in outcomes that cannot be used and may occur when unnecessary, duplicative studies are conducted, the wrong research questions are asked, flawed methodology is incorporated into design or study, or results that remain hidden from the public because of nonpublication. Furthermore, there is a mismatch between researchers, practicing clinicians, and patients with regards to relevance to clinical practice (*1*). To reduce the burden of research waste, increased investment in metabolism research must be weighed against the overall impact it will have on clinical practice. Efforts have been made to reduce research waste, such as performing systematic assessment of all existing evidence prior to conducting research. Despite this, concerns for research waste is still growing.

One way to evaluate the contribution of metabolism research is to examine citation counts as a proxy for importance, based on the assumption that highly cited articles contribute to advancing the field. The use of citations as a means of identifying those articles that have the most impact or value stems from endeavours to map the influence and spread of scientific innovation. Early work in this field originated, in part, from Garfield *et al.* who discussed “science mapping” using citations as a means to follow the dissemination of information within the scientific community (*2*). The theory behind this approach is that the number of citations a publication receives is directly proportional to the impact the publication has had on future research or knowledge (*2*, *3*). Thus, a lower rate of citation may suggest less impact on clinical practice and contribute to research waste (*1*). Here, we utilized citation rates in metabolism research over a 10-year period to improve our understanding of the journals that frequently publish these studies, and explore whether there are a high amount of publications that could be potentially contributing to research waste.

## Materials and methods

### Oversight and reporting

This study did not meet the regulatory definition of human subjects research as defined in 45 CFR 46.102 (d) and (f) of the Department of Health and Human Services’ Code of Federal Regulations, and, therefore, was not subject to Institutional Review Board oversight. We developed our methodology for data collection and comparison by consulting a previous study (*4*).

### Data source

We obtained citation rates within 5 years of publication of metabolism articles that were published between 2003-2013. We defined metabolism journals and articles as those that publish metabolism research, and used the keyword “metabolism” to identify such journals through The National Library of Medicine (NLM) catalogue. The NLM catalogue was used to identify metabolism journals, and PubMed was used to locate articles published by the included journals. We then used Scopus, a citation database by Elsevier, to obtain citation data *per* year for each article. The NLM catalogue search was conducted on 05/31/2019 and identified 114 total journals. We then excluded 3 journals for not being published in English, 34 for not being indexed in MEDLINE, 6 for not having enough publications *per* year (less than 20), and 4 for not publishing within the time period. After applying the exclusion criteria, we had a total of 67 journals to extract data from. Within each journal, we included original research articles, but excluded reviews, editorials, and letters. These forms of publication were excluded because reviews have been found to garner twice as many citations as original works. Other forms of publication, like editorials, usually do not contain original information, nor do they count in the denominator for the widely used impact-factor calculation (*5*, *6*).

Citation counts within five years of the date of publication were analysed because article citations have been found to peak within five years of publication (*7*). For this reason, the search was limited to studies published between 2003 and 2013. We included self-citations, as they may be used in follow-up research in the same field. While self-citations can inflate citation counts, it has been found to have a minimal effect on cumulative citations and H-index values in academic radiology research (*8*).

### Citation extraction and analysis

We transferred the citation information from Scopus into a pilot-tested Microsoft Excel file (Microsoft, Redmond, Washington) and counted the frequency of uncited, poorly cited, and well-cited articles *per* year. We defined articles as uncited if there were no citations, poorly cited if the article had one to five citations, and well cited if it received more than five citations within five years of publication. We generously chose 5 citations as the cut-off for being well cited because we wanted to focus our investigation on trends of poorly cited journals. This was also the cut-off used by Ranasinghe *et al*. in their analysis of cardiovascular journals (*4*). Using this cut-off allowed us to compare our results to theirs. To determine whether poorly cited articles were mainly found in certain journals, we assessed the proportion of poorly cited articles in each journal. Journals were classified as poorly cited if > 75% of their publications were poorly cited or uncited, well-cited if 26-75% were poorly cited or uncited, and highly cited if < 25% of their publications were poorly or uncited.

### Statistical analysis

All data calculations were made online in Google Sheets (Google LLC, Mountain View, California). This software allowed us to use formulas to analyse and sort raw data. We calculated the total number of articles *per* journal *per* year, the number and percentage of uncited, poorly cited, and well cited articles *per* year, as well as sorted the 67 journals into top 25% and bottom 25% based on how well cited their articles were. We also examined if open access journals represented the top 25%, middle 50%, or bottom 25% of journals. We then used the software to plot the data into graphs to demonstrate trends over time.

## Results

**Figure 1:**
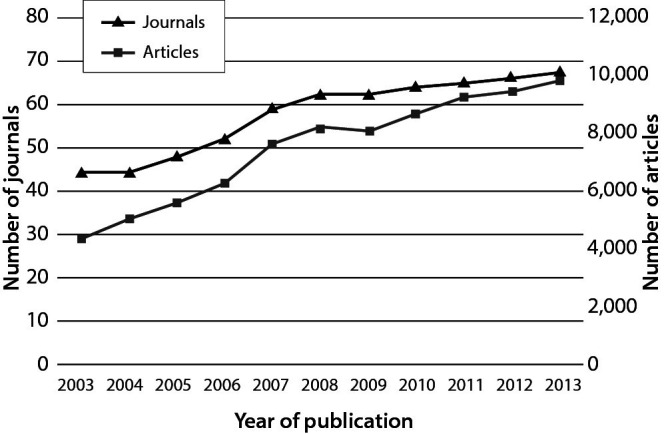
The yearly trend of metabolism journals and articles from 2003 to 2013.

Between 2003 to 2013, the total number of metabolism journals increased by 52%, from 44 to 67 journals (Figure 1). The number of articles published *per* year also increased, from 4314 to 9810, an increase of 127% (Figure 1). The total number of articles published from the included journals was 81,954.

**Figure 2:**
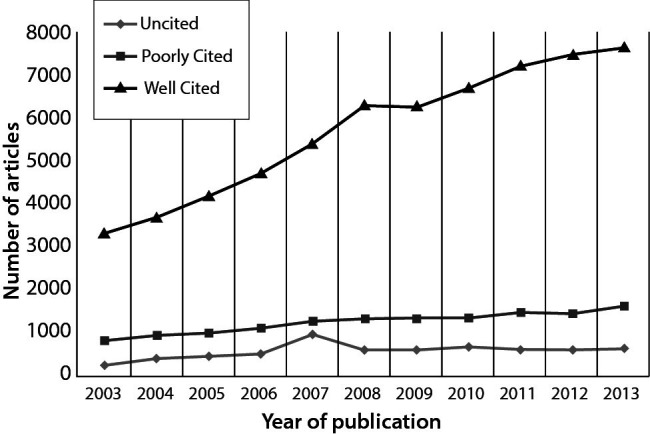
The number of uncited, poorly cited, and well-cited publications by year.

Of the 81,954 articles, 76% were well cited, 16.4% were poorly cited, and 7.2% were uncited within five years of publication (Figure 2). The percentage of well cited articles published *per* year increased from 77% in 2003 to 78% in 2013, poorly cited articles decreased from 19% in 2003 to 16% in 2013, and uncited articles increased from 5% in 2003 to 6% in 2013. The trend of the proportion of well cited, poorly cited, and uncited articles published each year remained relatively constant.

**Figure 3:**
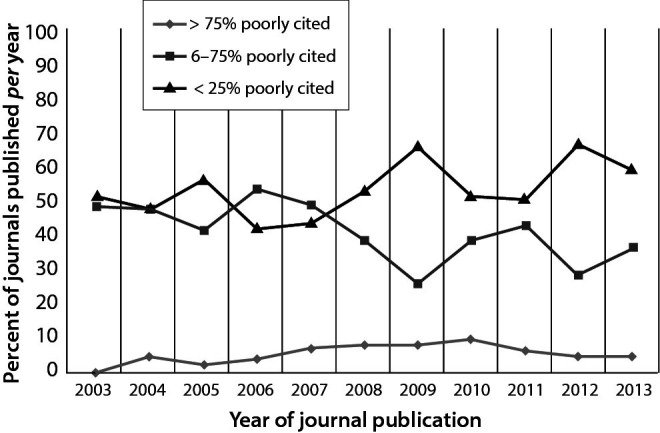
Comparison of journals based on percentage of poorly cited articles.

Journals were sorted by the proportion of articles that were poorly cited or uncited each year. The percentage of journals that were highly cited journals (< 25% poorly cited articles) increased from 51% in 2003 to 59% in 2013 (Figure 3). Well cited journals (between 26-75% poorly cited articles) decreased from 49% to 36%, poorly cited journals (> 75% poorly cited articles) increased from 0% to 5%.

We ranked the journals based on their proportion of well cited articles overall in the 10-year period and further sorted each journal into the top 25% or bottom 25% of the ranking. The top 25% of journals published 7635 articles in total, of which 19% were poorly cited or uncited. The bottom 25% of journals published 43,880 articles in total, of which 24% were poorly cited or uncited. The journals in the top 25% published 9% of the total articles and 8% of the total poorly cited and uncited articles. The journals in the bottom 25% published 54% of the total articles and 54% of the total poorly cited and uncited articles.

Of the 67 journals we analysed, eight were open access (12%). Five of the open access journals were placed in the top 25% for publishing highly cited articles, three were placed in the middle 50% of journals, and none were placed in the bottom 25%.

## Discussion

The findings of our citation analysis of metabolism studies published from 2003 to 2013 show that there is a high proportion of well cited articles in metabolism research, with the trend staying about the same over 10 years. These findings suggest that the majority of metabolism research is impactful and might not contribute to growing concerns for research waste. The total number of journals and articles published *per* year have increased. This is expected as research continues to grow and funding continues to increase. There is potential confounding since the increase in journals can naturally lead to an increase in articles. However, the percentage increase of articles greatly exceeds that of the increase in journals, indicating individual journals are publishing more articles overall. Our analysis of journals indicated that the proportion of highly cited, well cited, and poorly cited journals fluctuated every year and did not show significant change over the 10 years. The journals ranked in the bottom 25% published significantly more than those ranked in the top 25%. At first this seems that the bottom ranked journals have more quantity, and less quality. However, comparing the proportion of poorly cited articles the 2 groups of journals generated shows that the proportions are similar so they have similar quality. Lastly, we found that of the 8 journals that were open access, most of them were ranked in the top 25% for citation rate, and none were ranked in the bottom 25%. This suggests that publishing in an open access journal that provide better access to articles can increase the citation rate. This finding is in line with the findings of previous studies (*9*).

Our findings demonstrate important differences compared to overall trends noted in other fields. In a 2002-2006 analysis, it was found that only 45% of articles published in the top 4500 scientific journals were cited within the first 5 years of publication. Of the articles with citations, only 42% received more than one citation with a high proportion being self-citations (between 5-25%) (*10*). A 2015 analysis of the cardiovascular literature found that almost half of cardiovascular journal articles were poorly cited (less than 5 citations over 5 years), with an increase in proportion of poorly cited articles over time (*4*). However, our data showed less than 25% of journal articles were poorly cited, and this percentage has not changed significantly over 10 years. These findings contradict previous suggestions that “half of literature may be redundant as it is never cited” (*6*). Additionally, it disputes growing concerns over the largely unchecked expansion in publications resulting in what has been called “research waste” (*1*).

Here, we speculate that one potential reason for the discrepancy between our findings and those of previous studies is the exclusion of poor quality, predatory journals from our analysis. Predatory journals started by adopting the financial design of open access journals. Open access was designed to provide global access to published research by having the author pay a fee (*11*). The practice of open access has been corrupted by journals attempting to impersonate legitimate publishers. One could say these journals are predatory in nature because they often publish low quality research behind the guise of requiring a fee to be “open access” (*12*). A popular marketing tactic of some “predatory” journals is to solicit authors by obtaining their email addresses from PubMed or other bibliographic databases and then sending frequent invitations to publish with them (*13*). We believe the problem with these practices is two-fold. It draws authors in to a journal that will require a fee while forgoing the usual peer review process and leads to a publication that will rarely be indexed in MEDLINE/PubMed, leading to research waste (*14*). Additionally, the consequences associated with predatory publishing could be risky when skipping the peer review process. It could lead to the dissemination of false findings or the publication of studies with design flaws that would have been identified during the peer review process from reputable journals. During our data extraction, we first used NLM and PubMed to obtain a list of journals and articles to search on Scopus, then excluded 34 journals for not indexed in MEDLINE, and 9 more for not being in English or having enough publications. Compared to the study on cardiovascular journals done by Ranasinghe *et al.*, who obtained their list of journals and articles directly from Scopus and did not exclude journals based on MEDLINE, our study is less likely to include predatory and other poor quality journals (*4*). Had we not excluded journals based on our criteria, our results possibly would have been different.

The use of citation analysis to identify research “waste” has limitations, which have previously been described. A multitude of factors can influence citations, including language, length, number of authors, and type of article. Those that are written in English, longer in length, published in high impact journals, and are review articles or meta-analyses are more likely to be cited (*15*). Furthermore, citation rates could be skewed from self-citations, as discussed previously, and from same-journal citations. Another limitation of using citation analysis in a multidisciplinary field, such as metabolism, is that it includes basic science, behavioural health, public health, drug development, clinical medicine, and other disciplines. Additionally, we acknowledge that particular studies with low citation counts may provide meaningful contributions to the scientific corpus. Our intent was to use citation counts of those papers related to human metabolism on a broad level as a measure of overall influence, rather than to make specific claims regarding the influence of specific studies in this field. Despite the limitations of citation analysis, it remains a practical marker of overall usefulness of a publication, as the number of uncited and poorly cited works parallels the increase in yearly publications in other fields (*4*). With the increased focus on metabolism research secondary to its clinical impact, the need for continued assessment of research quality remains an ongoing focus in the era of research waste.

To conclude, our study found that over 75% of metabolism articles published between 2003-2013 are well cited within 5 years of their publication. Although the number of poorly cited and uncited articles increased over the 10-year period, their proportion compared to the total number of articles stayed relatively the same. However, we excluded many journals not indexed on MEDLINE. We therefore can only conclude that among journals indexed on MEDLINE, our findings suggest that based on citation rates alone, the phenomenon of research waste has not plagued the field of metabolism. Citation rates are also not the perfect indicator for research impact. Future research may be necessary to examine the citation rates of predatory and other poor quality journals excluded from our study and use other methods to evaluate the presence of research waste in the field of metabolism.

## References

[1] Chalmers I Glasziou P Avoidable waste in the production and reporting of research evidence. Lancet. 2009;374:86–9. 10.1016/S0140-6736(09)60329-9 19525005

[2] Garfield E, Malin MV, Small H. Citation Data as Science Indicators 1983. Available at: http://citeseerx.ist.psu.edu/viewdoc/summary?doi=10.1.1.15.4233. Accessed February 10th 2020.

[3] Wilken PH Elkana Y Lederberg J Merton RK Thackray A Zuckerman H Toward a Metric of Science: The Advent of Science Indicators. Soc Forces. 1979;57:1419 10.2307/2577293

[4] Ranasinghe I Shojaee A Bikdeli B Gupta A Chen R Ross JS Poorly cited articles in peer-reviewed cardiovascular journals from 1997 to 2007: analysis of 5-year citation rates. Circulation. 2015;131:1755–62. 10.1161/CIRCULATIONAHA.114.015080 25812573 PMC4560203

[5] Marashi SA On the identity of “citers”: Are papers promptly recognized by other investigators? Med Hypotheses. 2005;65:822. 10.1016/j.mehy.2005.05.003 15990244

[6] Weale AR Bailey M Lear PA The level of non-citation of articles within a journal as a measure of quality: a comparison to the impact factor. BMC Med Res Methodol. 2004;4:14. 10.1186/1471-2288-4-14 15169549 PMC434502

[7] Antunes AA Hoe to evaluate scientific production. Rev Col Bras Cir. 2015;42 Suppl 1:17–9. 10.1590/0100-69912015S01006 27437960

[8] Rad AE Shahgholi L Kallmes D Impact of self-citation on the H index in the field of academic radiology. Acad Radiol. 2012;19:455–7. 10.1016/j.acra.2011.11.013 22285543

[9] Chua SK Qureshi AM Krishnan V Pai DR Kamal LB Gunasegaran S The impact factor of an open access journal does not contribute to an article’s citations. F1000Res. 2017;6:208. 10.12688/f1000research.10892.1 28649365 PMC5464220

[10] Rawat S Meena S Publish or perish: Where are we heading? J Res Med Sci. 2014;19:87–9. 24778659 PMC3999612

[11] Laccourreye O Rubin F Maisonneuve H “Predatory” journals threatening the scientific medical press. Eur Ann Otorhinolaryngol Head Neck Dis. 2018;135:37–9. 10.1016/j.anorl.2017.08.003 28916412

[12] Beall J Predatory publishers are corrupting open access. Nature. 2012;489:179. 10.1038/489179a 22972258

[13] Dadkhah M Bianciardi G Ranking Predatory Journals: Solve the Problem Instead of Removing It! Adv Pharm Bull. 2016;6:1–4. 10.15171/apb.2016.001 27123411 PMC4845555

[14] Rydholm A Beware of predatory journals. Acta Orthop. 2017;88:576. 10.1080/17453674.2017.1387731 28990441 PMC5694797

[15] Seglen PO Why the impact factor of journals should not be used for evaluating research. BMJ. 1997;314:498–502. 10.1136/bmj.314.7079.497 9056804 PMC2126010

